# Attentional selection predicts rapid automatized naming ability in Chinese-speaking children with ADHD

**DOI:** 10.1038/s41598-017-01075-x

**Published:** 2017-04-20

**Authors:** Encong Wang, Meirong Sun, Ye Tao, Xiaoyi Gao, Jialiang Guo, Chenguang Zhao, Hui Li, Qiujin Qian, Zhanliang Wu, Yufeng Wang, Li Sun, Yan Song

**Affiliations:** 1 State Key Laboratory of Cognitive Neuroscience and Learning & IDG/McGovern Institute for Brain Research, Beijing Normal University, Beijing, China; 2 Peking University Sixth Hospital/Institute of Mental Health, Beijing, China; 3 National Clinical Research Center for Mental Disorders (Peking University Sixth Hospital), Key Laboratory of Mental Health, Ministry of Health (Peking University), Beijing, China; 4 Faculty of Education, Beijing Normal University, Beijing, China; 5 Center for Collaboration and Innovation in Brain and Learning Sciences, Beijing Normal University, Beijing, China

## Abstract

Children with attention-deficit/hyperactivity disorder (ADHD) are reported to have a significantly higher risk of showing reading difficulties or disorders. Here, we aimed to identify the relationship between electroencephalographic (EEG) marker of spatial attention and reading ability in Chinese children with ADHD. First, we demonstrated that rapid automatized naming (RAN) is a strong predictor of reading ability in Chinese-speaking children. Then, EEG data of 9-to 15-year-old children with ADHD (n = 38) and typically developing (TD) controls (n = 36) were collected while the children performed a classical visual search task. Children with ADHD showed slower RAN speed than TD children. For event-related potentials (ERPs), children with ADHD showed a reduced target-evoked N2pc component, which predicted their poorer RAN performance. However, in TD children the early occipital P1 amplitude was negatively correlated with their RAN performance. The correlation between decreased N2pc and poor RAN performance in children with ADHD suggests that their reading problems may in part be due to impaired attentional selection. In contrast, in TD children, development in early visual processing co-occurs with improvements in reading ability.

## Introduction

Attention-deficit/hyperactivity disorder (ADHD), one of the most prevalent neuro-developmental disorders diagnosed in childhood and adolescence, is characterized by inattention and/or hyperactivity/impulsivity. In addition to these two core ADHD symptom dimensions, children with ADHD are at significantly higher risk of reading and/or spelling difficulties or disorders^1–4^. Approximately 25% to 40% of children with ADHD also meet criteria for reading disorder^5^. This may reflect the effects of attentional problems on reading performance. Previous studies have shown that ADHD and reading disorder may share a common cognitive deficit in visual processing, primarily due to common genetic influences^6, 7^. However, the neural substrate underlying this phenomenon has yet to be characterized. In the present study, we report a first attempt to identify the relationship between electroencephalographic (EEG) markers of visual spatial attention and reading ability in children with ADHD.

Although visual spatial attention appears to have little to do with reading ability, recent reading theories have highlighted that reading involves multiple linguistic, visual, as well as attentional processes^8^. Several recent twin studies indeed demonstrate a strong association between reading and subjective inattention symptoms^6, 7, 9^. More importantly, studies in both adults and children with reading difficulties have revealed deficits in visual-spatial performance^10, 11^. These studies suggest that a weakness in visual-spatial attention, independent of language, could cause reading difficulty^12^.

Previous behavioral studies reported that individuals with ADHD have unimpaired selective attention^13, 14^. However, a series of recent EEG studies using spatial cueing paradigms found abnormal alterations in posterior alpha and frontal theta activity as well as their functional disconnection in response to a cue in children with ADHD^15–17^. These EEG results imply the possible occurrence of spatial attention impairments in ADHD. For event-related potentials (ERPs), one particular ERP component known as the N2pc (posterior contralateral N2) is a well-characterized index of covert visual attentional selection^18–21^. Recently, it was reported that N2pc latency was prolonged in ADHD adults^22^, and N2pc amplitude was reduced in children with ADHD^23^. More importantly, there were significant correlations between N2pc parameters and ADHD symptom severity. These studies imply that these signals of visual-spatial attention may serve as potential candidates for neurophysiological markers of ADHD^24^, providing strong neurophysiological evidence that specific processes (e.g., attentional selection) in visual-spatial attention are impaired in ADHD patients. Moreover, recent ERP evidence also suggests that reading ability is positively correlated with the amplitude of the P1 component in healthy adults, suggesting that reading has a substantial impact on the magnitude, precision, and invariance of early visual processing, as early as ~100–150 ms in the visual cortex^25^.

In order to evaluate the reading ability of children, we used a classical rapid automatized naming (RAN) test, which is a well-known independent predictor of reading fluency that discriminate between dyslexic and nondyslexic reading groups^26–28^. RAN can well explain individual differences in reading ability, and at least 104 studies published from 1990 to 2009 have used RAN as a measure of reading skills^29^. RAN has also been linked to reading development and impairment both in English^30^ and in Chinese^31, 32^. Among commonly used items (digits, letters, colours, or objects), naming fluency for alphanumeric stimuli (digits and letters) in particular remains a strong predictor of reading ability^33^ that persists into adulthood^34^. This tendency has also been confirmed in Chinese readers^35, 36^.

In the present study, we first confirmed previous results demonstrating that RAN is a powerful predictor of reading fluency for Chinese-speaking children in Experiment 1. We then recorded ERPs from Chinese-speaking children (including children with ADHD and typically developing children) when they were performing a classical visual search task in Experiment 2. We predicted that the relationship between reading ability and the biomarker of attentional selection (N2pc) might differ between children with ADHD and typically developing (TD) children, suggesting that visual-spatial attention affects reading through different patterns in ADHD. Additionally, children with ADHD and TD children might also show different associations between reading and early visual processing. Here, the relationship between ERP components and RAN performance was analyzed for the two groups of children separately.

## Results

### Behavior in RAN test in Experiment 1

**Figure 1:**
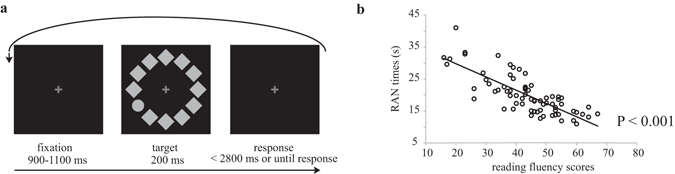
(**a**) Trial sequence of the visual search paradigm. (**b**) Scatter plot depicts the relationship between RAN times and reading fluency scores in Chinese-speaking children.

First, the relationship between RAN and reading fluency ability in Chinese children was tested in Experiment 1. Naming speed for all children was 20.0 ± 6.2 s (mean ± standard error). Reading fluency scores were 43.5 ± 11.7 sentences. There was a highly significant correlation between Digit RAN and reading fluency (r = −0.782, P < 0.001; Fig. 1b). After controlling for children’s age, this correlation still remained highly significant (r = −0.504, P < 0.001). This result is consistent with previous studies showing that Digit RAN is a good predictor of reading fluency in Chinese-speaking children^34^.

### Behavior in RAN test in Experiment 2

Then, the same Digit RAN task was used as a measurement of reading ability in Experiment 2 to evaluate the reading ability of ADHD and TD children. None of the children in Experiment 2 had participated in Experiment 1. As illustrated in Table 1, children with ADHD were significantly slower in performing the RAN task than TD children (ADHD: 22.2 ± 6.9 s; TD: 16.9 ± 3.6 s; t(67) = 4.132, P* < *0.001, Cohen’s d = 0.98, Power = 0.98), suggesting that reading ability is impaired in children with ADHD. Additionally, age was negatively correlated with performance on the RAN task in the ADHD group (r = −0.563, P < 0.001), but not in the TD group (r = 0.082, P = 0.656), which may be related to different developmental patterns of reading between the two groups. IQ and ADHD symptom severity were not significantly associated with performance on the RAN task for either group.

**Table 1:** Sample characteristics and behavioral results in the ADHD and TD groups.

	ADHD (n = 38)		TD (n = 36)		Comparison	
	M	SD	M	SD	t/χ^2^	P values
Number of males	32	—	27	—	0.970	0.325
Child age (years)	11.6	1.7	11.2	1.4	1.079	0.284
Full scale IQ	108	11	112	15	−1.574	0.120
**ADHD-RS**						
Inattention	26.9	2.7	12.5	2.5	20.104	<0.001
Hyperactivity/Impulsivity	19.7	4.8	11.2	3.2	8.024	<0.001
**Behavioral Results**						
RAN times (s)	22.2	6.9	16.9	3.6	4.132	<0.001
**Spatial attention task**						
Median RTs (ms)	638.2	137.4	573.0	127.5	2.114	0.038
Accuracy (%)	93.4	7.3	95.1	4.6	−1.252	0.215
RT_SD_ (ms)	206.1	76.6	149.5	55.0	3.658	<0.001

ADHD: attention-deficit/hyperactivity disorder; TD: typically developing; ADHD-RS: ADHD-rating scales of behavioral symptoms; RAN: rapid automatized naming; RTs: reaction times; RT_SD_: variance of reaction times.

### Behavior in visual search task during EEG recording in Experiment 2

Children with ADHD were overall slower (t(72) = 2.144, P = 0.038, Cohen’s d = 0.49, Power = 0.55) and more variable in their RTs (t(72) = 3.658, P < 0.001, Cohen’s d = 0.85, Power = 0.95; Table 1). Accuracy did not significantly differ between the two groups (t(72) = −1.252, P = 0.215, Cohen’s d = 0.29, Power = 0.23). These findings are consistent with previous studies showing that slower RTs are reliably found in children as well as in adults with ADHD^12, 21^. The RT cost might reflect either a genuine (age-independent) slowing or a compensatory speed-accuracy trade-off strategy in ADHD.

### ERPs in visual search task in Experiment 2

There was no correlation between ERPs and IQ (Pearson’s r = from 0.002 to 0.199, Ps > 0.231). We focused on the relationship between ERPs and reading ability in each group.

### P1 component

**Figure 2:**
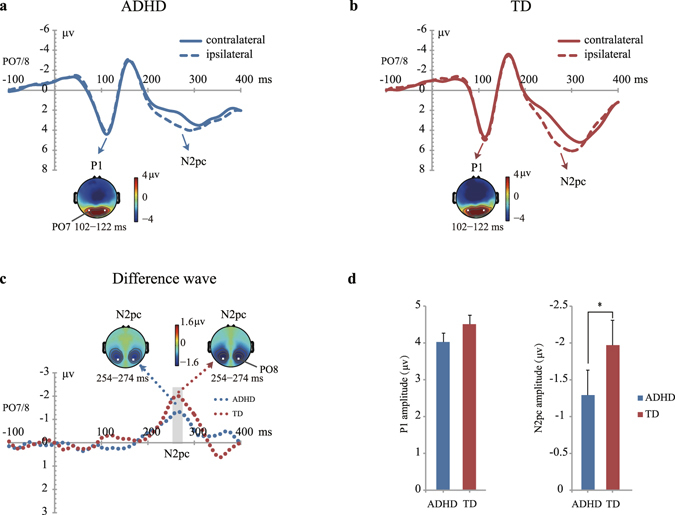
Grand averaged event-related potentials (ERPs) at contralateral and ipsilateral electrode sites (averaged over PO7 and PO8) relative to the target in children with ADHD (**a**) and TD children (**b**). Grand average difference waveforms (**c**) obtained by subtracting ipsilateral from contralateral waveforms. Topographic scalp maps show the distribution of P1 and N2pc components. The schematic illustration in (**d**) shows the mean voltages (and SE) of P1 and N2pc of ADHD and TD groups.

Figure 2a and b show ERP waveforms in response to visual search arrays from electrodes over the visual cortex contralateral and ipsilateral to the target (PO7 and PO8). As can be seen clearly, a robust P1 component was elicited during 100–130 ms post-stimulus in both groups. For the P1 amplitudes (102–122 ms), neither the “Group” nor the “Contra-Ipsi” effect reached significance (“Group”: F(1,72) < 1, P = 0.634, η_p_^2^ = 0.003, Power = 0.07; “Contra-Ipsi”: F(1,72) < 1, P = 0.825, η_p_^2^ = 0.001, Power = 0.06; Fig. 2d). We also analyzed P1 latency and no significant difference was found between the two groups (“Group”: F(1,72) < 1, P = 0.320, η_p_^2^ = 0.014, Power = 0.17; “Contra-Ipsi”: F(1, 72) < 1, P = 0.558, η_p_^2^ = 0.005, Power = 0.09).

We further found that age was negatively correlated with P1 amplitudes for both TD children (r = −0.378, P = 0.023) and children with ADHD (r = −0.574, P < 0.001). Age was also negatively correlated with P1 latency for both the TD (r = −0.344, P = 0.040) and the ADHD group (r = −0.405, P = 0.012). These results are consistent with prior work reporting age-related reductions in P1 amplitude and latency^37, 38^, which may correspond to increasing automaticity of visual processing throughout childhood.

**Figure 3:**
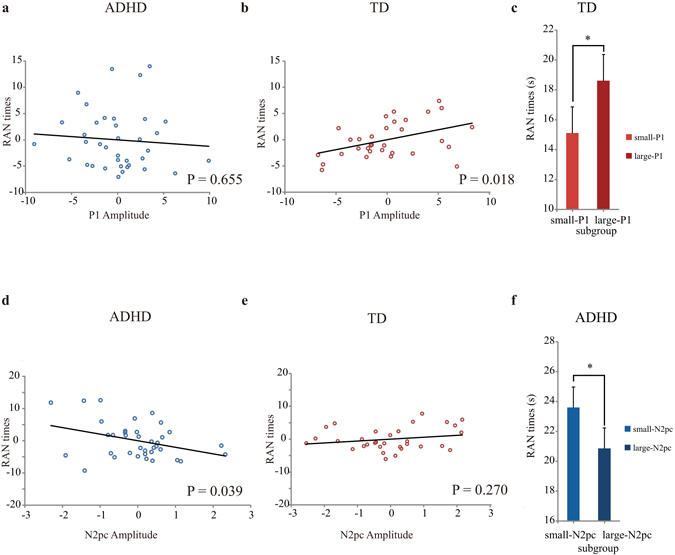
Scatter plots depict regression results between P1 amplitude and RAN times in children with ADHD (**a**) and TD children (**b**) after controlling for age and IQ. The schematic illustration in (**c**) shows mean times (and SE) of RAN for small-P1 and large-P1 subgroups for TD children. Scatter plots depict regression results between target-elicited N2pc amplitude and RAN times in children with ADHD (**d**) and TD children (**e**) after controlling for age, IQ and N2pc latency. The schematic illustration in (**f**) shows mean times (and SE) of RAN for small-N2pc and large-N2pc subgroups for children with ADHD.

To test whether P1 was linked to children’s reading ability, we constructed a multiple regression model with P1 (P1 amplitude, P1 latency) for RAN performance for each group. The ERP measures were entered as continuous independent variables. We included age and IQ as continuous covariates of no interest. As illustrated in Supplementary Table S1, the strongest relationship for the TD group was observed for P1 amplitude; smaller P1 amplitudes predicted faster RAN speeds (b = 0.461, P = 0.018; Fig. 3b) for the TD group. On the other hand, no significant effect was found for children with ADHD (Fig. 3a).

To further confirm the different correlations between the two groups, we tested the P1 amplitude-related regression slope for RAN in another multiple linear regression for the whole sample, by setting ADHD status as a dummy predictor (ADHD = 0, TD = 1). If the betas for P1 amplitude were indeed differential for ADHD and TD regarding RAN, the interaction between P1 amplitude and Group would be significant. The results showed that P1 amplitude can predict RAN times (b = 0.345, P = 0.028). Additionally, the P1 amplitude-related regression slope on RAN differed for children with ADHD and TD as indicated by a significant interaction between P1 amplitude and Group (b = −0.420, P = 0.003).

To rule out that the correlation presented in Fig. 3b is driven by outliers, a further robust regression analysis was performed, and the result supported the existence of this significant correlation (b = 0.457, P = 0.016). To help visualize the relationship between reading and P1, we then sorted the TD children into two subgroups based on a median split of the P1 amplitude and constructed separate reading performance for each subgroup. The small-P1 subgroup showed significantly faster RAN speed compared with the large-P1 subgroup (F(2, 29) = 11.533, P = 0.002, η_p_^2^ = 0.285, Power = 0.87; Fig. 3c), further supporting the significant correlation between P1 amplitude and reading ability in TD children.

### N2pc component

A reliable N2pc component was elicited during 200–300 ms post-stimulus in both groups (Fig. 2a,b), suggesting that the target appearance evoked a contra-lateralized neural effect in children. This can also be observed in the contralateral-minus-ipsilateral difference waveforms and the topographic maps shown in Fig. 2c. The scalp distribution is qualitatively similar to that observed in prior N2pc studies in adults^18–20, 39, 40^.

To assess the statistical significance of this target contra-lateralized effect, the amplitude of N2pc was measured as the mean value of a 20-ms window centered at the peak of the difference wave (254–274 ms). One-sample t-tests revealed that the N2pc amplitude was significantly different from zero at PO7/PO8 for children with ADHD (t(37) = −5.548, P < 0.001, Cohen’s d = 0.90, Power = 1) and TD children (t(35) = −8.199, P < 0.001, Cohen’s d = 1.37, Power = 1), respectively. We then focused on the N2pc difference between the two groups. N2pc peak latency was nearly identical for both the ADHD and TD group (ADHD: 250 ± 34 ms; TD: 259 ± 29 ms; t(72) = −1.208, P = 0.231, Cohen’s d = 0.28, Power = 0.23). However, the amplitude of N2pc in children with ADHD was smaller than that in the TD group (t(72) = 2.023, P = 0.047, Cohen’s d = 0.47, Power = 0.52; Fig. 2d). We further found a significant correlation between N2pc amplitude and N2pc peak latency for the ADHD group (r = 0.499, P < 0.001). That is, larger N2pc amplitudes were associated with longer N2pc latencies, indicating that there may be a time compensation mechanism for children with ADHD to deploy more attentional resources to the target. On the other hand, no significant correlation between N2pc amplitude and its latency was found for the TD group (r = 0.004, P = 0.980). Age was not significantly correlated with N2pc for either group.

We then tested whether N2pc was linked to children’s reading ability. Since the N2pc amplitude was correlated with its latency in children with ADHD, we regressed out the variance of the N2pc latency from that of the N2pc amplitude to isolate the N2pc amplitude effect in the following analyses. We constructed a multiple regression model with N2pc amplitude for RAN performance for each group. After controlling for the confounding factors of age, IQ, and N2pc latency (Supplementary Table S1), the residual N2pc amplitude was negatively correlated with RAN times in children with ADHD (b = −0.293, P = 0.039; Fig. 3d), i.e., smaller N2pc amplitudes predicted longer RAN times. It should be noted that the correlation between N2pc amplitude and RAN did not reach significance level for either group (ADHD: r = 0.017, P = 0.920; TD: r = −0.270, P = 0.135) when N2pc latency was not controlled. On the other hand, we did not observe significant effects in TD children (Fig. 3e).

To rule out that the correlation presented in Fig. 3d is driven by outliers, a further robust regression analysis was carried out; the correlation still reached significance (b = −0.362, P = 0.035). To help visualize the relationship between RAN performance and N2pc, we then sorted the children with ADHD into two subgroups based on a median split of the N2pc amplitude. The small-N2pc subgroup was found to have significantly slower RAN performance than the large-N2pc subgroup (F(2,33) = 6.899, P = 0.013, η_p_^2^ = 0.173, Power = 0.65; Fig. 3f), further supporting the significant correlation between N2pc amplitude and RAN in children with ADHD.

### Further analyses

ADHD is a highly heterogeneous disease. In the following analyses, data from 13 children with ADHD comorbid learning disorder were excluded, and the data from the remaining 25 ‘pure’ children with ADHD and 36 TD children were re-analyzed. Both the behavioral results and ERPs results were similar to the results for the whole sample, as presented above. Children with ADHD were still slower (t(59) = 1.976, P = 0.053, Cohen’s d = 0.51, Power = 0.48) and more variable in their RTs (t(59) = 3.020, P = 0.005, Cohen’s d = 0.81, Power = 0.87). P1 amplitude predicted RAN only in TD children (b = 0.461, P = 0.018) whilst the N2pc amplitude predicted RAN only in children with ADHD (b = −0.256, P = 0.09).

## Discussion

Reading is a unique, cognitive human skill crucial to life in modern societies. Researchers have hypothesized that deficits in visual spatial attention contribute to reading difficulties in children^12, 34, 41, 42^. On the basis of behavior alone, however, it is difficult to determine whether attentional processes or other higher-order cognitive processes underlie the relationship between attentional deficits and reading difficulties. ERP is a promising tool for multilevel investigations due to its high temporal resolution^43^. Therefore, we addressed this enduring issue by examining rapid naming performance and ERPs in a classical pop-out visual search task in both TD children and children with ADHD. In the present study, we observed distinct correlation patterns between ERPs and reading performance for children with ADHD and TD children. We found, in children with ADHD only, that the aberrant modulation amplitude in the target-evoked N2pc and RAN performance were correlated, indicating that poor reading ability in ADHD is at least in part related to attentional problems, possibly arising from deficits in attentional selection. In contrast, in TD children, early visual processing was correlated with RAN performance, suggesting that development in early visual processing co-occurs with improvements in reading ability during normal childhood.

Learning to read represents opportunistic training of neural mechanisms underlying a range of cognitive, perceptual, and motor skills that evolved for other purposes. One of the most relevant, neurocognitive functions used for reading may be the attentional mechanism used in visual search^44^, which is needed for recognition of one item at a time and also for ‘binding’ different objects. Therefore, the time required by children to learn to read effectively may be directly related to the need for training of visual search mechanism^12^.

A fundamental question posed by these studies is what role spatial attention in visual search plays during reading development. In the present study, we found that the unique circle target singleton elicited an obvious N2pc component in both ADHD and TD children, reflecting attentional selection of task-relevant stimuli. However, compared with TD children, children with ADHD showed much smaller target-evoked N2pc, which has been deemed an electrophysiological marker for visual attentional selection. More importantly, after controlling for the effect of N2pc latency on its amplitude, the reduced N2pc amplitude for children with ADHD predicted their prolonged response times in RAN, which is a well-established, powerful test of children’s reading ability. Our results provide strong neurophysiological evidence that attentional selection is impaired in children with ADHD, and this impairment may be closely related to poor reading ability.

The absence of group differences in the early P1 response to visual stimuli found here is consistent with results from one recent study^17^ suggesting that children with ADHD have unimpaired visual sensory coding. More importantly, both P1 amplitude and latency were negatively correlated with age in children with ADHD in this study. This developmental pattern of P1 in children with ADHD is similar to TD children in our present study as well as previous literature^37, 38^. The age-related reductions in P1 latency may reflect maturation in the speed of stimulus processing, and the age-related reductions in P1 amplitude may reflect synaptic pruning throughout childhood^38, 45^. This ERP maturation might reflect a general developmental trend for both TD and children with ADHD, which may be due to overall sensory and cognitive development^46^.

Most importantly, our results further demonstrate that development of early visual processing can predict reading ability in TD children (i.e., better readers exhibit smaller P1 amplitudes), but not in children with ADHD. This finding was confirmed by a significant interaction effect between the P1 amplitude and Group for the whole sample as well as the different betas for separate groups. Our present findings seem to contrast with those of a recent study conducted by Pegado *et al*.^25^, who found that poor adult readers have smaller P1 amplitudes. The critical difference between Pegado *et al*.’s study and our current work, however, is that the participants in Pegado *et al*.’s study were healthy adults (32–68 years old) with different levels of literacy, including completely illiterate subjects, early-schooled literate subjects, and adults who learned to read in adulthood. Therefore, the P1 effect observed by Pegado *et al*. reflects the impact of literacy on early visual processing^25^. In contrast, participants in the present study were two groups of children with equal levels of formal early-schooled literacy training. The fact that we did not find significant group differences in early P1 responses is thus compatible with Pegado *et al*.’s finding. Meanwhile, the present study demonstrates that, after controlling for the impact of literacy training, development in early visual processing co-occurs with improvements in reading ability during normal childhood.

In conclusion, our findings suggest that a dysfunction of the neural mechanism that modulates attentional selection plays a key role in reading problems in children with ADHD. In contrast, in control children, attentional selection does not seem to be the limiting factor for reading performance; development of early visual processing, however, seems to be involved in determining behavioral reading performance.

Although the present results were obtained by controlling for age and IQ, there may be other factors affecting the relationship between reading performance and spatial attention. First, given the contribution of skills tested in the RAN task to reading ability and distinctive orthographic features of Chinese, it is important to investigate whether our conclusions can be generalized to phonetic alphabetic languages, such as English, in further studies. Second, it should be noted that ADHD is a highly heterogeneous disease^47^. A limitation of the present study is that we could not differentiate between ADHD with dyslexia and ‘pure’ ADHD. Therefore, future studies have to further elucidate the relationship between reading ability and attentional deficit in ADHD. We are currently utilizing several more complex reading measures, for example, the Standardized Chinese Character Recognition Test^48^, to precisely assess the different aspects of children’ reading abilities. These measurements can help us to distinguish several phenotypes of children, especially children with comorbid ADHD and dyslexia. Last, it is important to investigate whether our conclusions can be generalized to all phenotypes of ADHD in further studies.

## Materials and Methods

### Experiment 1: RAN predicts reading fluency in Chinese-speaking children

#### Participants

In Experiment 1, seventy-one children (43 males, age 8–14 years) with normal or corrected-to-normal vision were recruited. This study was approved by the Beijing Normal University Institutional Review Board. The complete study was carried out in accordance with the ethics standards of the Declaration of Helsinki. Informed consents were obtained from all children as well as their parents.

#### RAN test

Children were administered a Digit RAN task, which is a particularly strong indicator of literacy development in Chinese children, to assess their ability in rapid naming. It consists of 5 digits (2, 4, 6, 7, 9) repeated 10 times in a random sequence, yielding 50 stimuli presented in 5 rows of 10 items on a sheet. Children were required to name the digits as accurately and quickly as possible, and the experimenter measured each naming time with a stopwatch. Children went through two trials, and the average time across trials was used as naming speed for subsequent analyses.

#### Reading fluency test

This test was a reading comprehension test which was composed of 95 sentences^49, 50^. Each sentence was paired with five multiple-choice pictures. Children were asked to silently read each sentence in isolation and then select, from five pictures, the one that best illustrated the meaning of the sentence. The children were encouraged to complete as many paragraphs as possible within a seven-minute’ time period. The total number of sentences that participants could correctly understand determined their performance score. This task requires rapid retrieval and retention of lexical information and construction of sentential representation. All sentences in this test are composed of high-frequency characters and words; it is therefore very easy for participants to grasp the meaning of each sentence. We used this test to evaluate children’s reading speed as the total number of sentences they could accomplish.

### Experiment 2: N2pc predicts RAN performance in Children with ADHD

#### Participants

In Experiment 2, ninety Chinese-speaking children (48 with ADHD, 65 males, ages 9–15 years) with normal or corrected-to-normal vision were recruited. None had participated in Experiment 1. We evaluated children for ADHD and other psychiatric disorders through a semi-structured diagnostic interview with the primary caretaker (usually the mother) and a direct interview with the child. For strict diagnosis, children with ADHD were diagnosed by two trained pediatric psychiatrists, one of whom was a senior trained psychiatrist according to the Clinical Diagnostic Interviewing Scales (CDIS). Diagnosis of ADHD was based on DSM-IV criteria (see Supplementary information for ADHD inclusion criteria). TD children were recruited from primary and high schools near the Institute of Mental Health, Peking University, and were subjected to identical screening procedures as children with ADHD. TD children did not fulfill the criteria of any neurological or psychiatric disorder such as ADHD, dyslexia, autism spectrum disorder, tic disorder or obsessive-compulsive disorder.

Data from 8 participants were discarded because of the high ratio of noise in their EEG signals. Data from another 8 participants were excluded due to excessive vertical or horizontal eye movements (see Supplementary information for objective exclusion criteria). Therefore, 38 drug-naive children with ADHD (32 males) and 36 TD children (27 males) were included in the final ERP component analysis. Age, IQ, and gender ratios were matched between the two groups (Table 1). Among the 38 children with ADHD, 13 children with ADHD had a comorbid learning disorder (see Supplementary information for criteria of learning disorder). Informed consents were obtained from all children as well as their parents. The complete study was approved by the Ethics Committee of the Peking University Institute of Mental Health in accordance with the Declaration of Helsinki.

#### Visual search paradigm

All children participated in a visual search task, in which they were required to find a target shape within an array of distractors (Fig. 1a). The stimulus array consisted of a circle (target) and 11 diamonds. The target was randomly located in either the right visual field (2 or 4 o’clock) or left visual field (8 or 10 o’clock) with equal probability. Each trial started with the presentation of a fixation cross at the center of the screen for 900–1,100 ms, followed by the presentation of the circular search array for 200 ms. The fixation cross remained on the screen at all times until a response was detected or until up to 3 s. A total of 8 successive blocks of 30 trials per block were run in Experiment 2, lasting about 17 minutes. Children had unlimited free rest periods between blocks in the ERP sessions. While performing the visual search, participants were instructed to maintain their gaze at fixation, and report the position of the target circle (upper or lower), but to ignore the diamonds.

#### RAN test

In Experiment 1, we demonstrated that Digit RAN is a good predictor of reading fluency in Chinese-speaking children. Therefore, the same Digit RAN task was used in Experiment 2 to evaluate reading ability of ADHD and TD children. Specifically, 37 of the 38 children with ADHD and 32 of the 36 TD children participated in the Digit RAN test.

#### ERP recording and analysis

Continuous EEG was recorded from 128 channels (HydroCel Geodesic Sensor Net, Electrical Geodesics, Inc., Eugene, OR) simultaneously with Net Station EEG Software, while children were performing a visual search task. All electrodes were physically referenced to Cz (fixed by the EGI system) and were then re-referenced off-line to the average of the left and right mastoids. The impedance of all electrodes was kept below 50 kΩ during data acquisition. The EEG was amplified with a band pass of 0.01–400 Hz (half-power cutoff) and digitized online at 1,000 Hz.

Offline EEG processing and analyses were performed using custom MATLAB (Mathworks) scripts and functions from the EEGLAB environment^51^. The EEG data were band-pass filtered (half-power cutoff at 1–40 Hz) with a roll-off of 12 dB/octave, and were then re-referenced to the average of the left and right mastoid channels. Electrodes containing excessive artifacts or high-amplitude, high-frequency muscle noise were excluded from further analyses. Data from all task blocks were concatenated to form a continuous time series. This time series was subsequently inspected for outlier epochs encompassing gross movements and muscle artifacts, and such time series were removed. The trimmed data were then decomposed into maximally independent component processes using temporal ICA decomposition via extended infomax. Components associated with vertical eye movements were visually identified and removed, according to their spatial, spectral, and temporal properties. The data were then segmented relative to stimulus onset (−200 to 600 ms), and the baseline preceding the stimulus (−200 to 0 ms) was subtracted. Epochs were sorted according to target visual field (left, right) for each group of children. To further control for horizontal eye movements, we rejected all segments with signals exceeding ±50 μV at the difference waves of electrodes F9/10 during 200–400 ms before ERP averaging. To further control for eye blinking or closing during the presentation of the stimulus, we also rejected all segments with signals exceeding ±70 μV at electrodes F1/2 during 0–200 ms from the original segmented data before ICA analyses. Epochs contaminated by incorrect responses, responses faster than 200 ms, or responses slower than 2,000 ms were also excluded from the ERP averages. Last, to assess whether any systematic horizontal EOG activity was present in the remaining data, we computed averaged F9/10 waveforms for left and right target trials. In all participants, residual activity was less than 3.2 μV^52^. An average of 21.3% of trials were rejected on the basis of artifacts for the final set of participants. There was no significant difference between the number of valid trials (range 169–190) for the ADHD and TD group (Fs < 0.245, Ps > 0.622, η_p_^2^s < 0.002).

P1 and N2pc were measured at the PO7/8 electrode sites. For each subject, P1 peak latencies were determined as the maximum deflection in the time window of 70–150 ms. N2pc peak latencies were determined as the minimum deflection of difference waveform in the time window of 180–300 ms for each subject. The amplitudes of P1 and N2pc were measured as the mean values of 102–122 ms and 254–274 ms, respectively. P1 was subjected to a repeated measures ANOVA, in which the within-group factor was “Contra-Ipsi” (contralateral vs. ipsilateral electrode, relative to the target), and the between-group factor was Group (TD vs. ADHD). The degrees of freedom were adjusted using Greenhouse-Geisser corrections when appropriate. Comparisons of N2pc at the PO7/8 electrode sites as well as behavioral performance (RT, accuracy, RT_SD_) between the two groups were made using two-tailed independent samples t-tests.

## Electronic supplementary material

Attentional selection predicts rapid automatized naming ability in Chinese-speaking children with ADHD
