# Supplementary material for: Attentional selection predicts rapid automatized naming ability in Chinese-speaking children with ADHD
Source: Sci Rep. 2017 Apr 20;7:939. doi: 10.1038/s41598-017-01075-x (PMC5430513; doi:10.1038/s41598-017-01075-x)
Supplement: Supplementary file 1 — Attentional selection predicts rapid automatized naming ability in Chinese-speaking children with ADHD [file 41598_2017_1075_MOESM1_ESM.pdf]

Supplementary information

Attentional selection predicts rapid automatized naming ability in Chinese-speaking children with ADHD

Encong Wang[1,2,3,+], Meirong Sun[1,+], Ye Tao[1,+], Xiaoyi Gao[4], Jialiang Guo[1], Chenguang Zhao[1], Hui Li[2,3], Qiujin Qian[2,3], Zhanliang Wu[2,3], Yufeng Wang[2,3], Li Sun[2,3,*], and Yan Song[1,5,*]

[1]State Key Laboratory of Cognitive Neuroscience and Learning & IDG/McGovern Institute for Brain Research, Beijing Normal University, Beijing, China

[2]Peking University Sixth Hospital / Institute of Mental Health, Beijing, China

[3]National Clinical Research Center for Mental Disorders (Peking University Sixth Hospital), Key Laboratory of Mental Health, Ministry of Health (Peking University), Beijing, China

[4]Faculty of Education, Beijing Normal University, Beijing, China

[5]Center for Collaboration and Innovation in Brain and Learning Sciences, Beijing Normal University, Beijing, China

+ These authors contributed equally to this work.

Materials and Methods

Experiment 2: N2pc predicts RAN performance in Children with ADHD

We evaluated children for ADHD and other psychiatric disorders through a semi-structured diagnostic interview with the primary caretaker (usually the mother) and a direct interview with the child. For strict diagnosis, children with ADHD were diagnosed by two trained pediatric psychiatrists, one of whom was a senior trained psychiatrist according to the Clinical Diagnostic Interviewing Scales (CDIS). Diagnosis of ADHD was based on DSM-IV criteria. All children with ADHD in this study met the following inclusion criteria: 1) meeting the diagnostic criteria of the Diagnostic and Statistical Manual of Mental Disorders, Fourth Edition (DSM-IV) for ADHD; 2) right handedness; 3) normal or corrected-to normal visions; 4) no history of taking stimulant drugs to treat ADHD symptoms; 5) exclusion of organic diseases, schizophrenia, mood disorder, autism spectrum disorders or epilepsy; and 6) full scale intelligence quotient (IQ) of at least 80 on the Chinese Wechsler Intelligence Scale for Children (C-WISC).

Data from 8 participants (5 with ADHD, 3 males) were discarded because of the high ratio of noise in their EEG signals (more than 10 bad electrodes that contained excessive artifacts or high-amplitude, high-frequency muscle noise exceeding 50% of the total recording time). Data from another 8 participants (5 with ADHD, 3 males) were excluded due to excessive vertical or horizontal eye movements (more than 50% of trials rejected or residual activity at electrodes F9/10 more than 2 μV). Therefore, 38 drug-naive children with ADHD (32 males) and 36 TD children (27 males) were

included in the final ERP component analysis. Age, IQ, and gender ratios were matched between the two groups (Table 1).

Through the Clinical Diagnostic Interviewing Scales (CDIS) according to DSM-IV criteria, we could assess both ADHD symptoms and associated disorders for each child, such as mood disorder, obsessive-compulsive disorder, tic disorder and learning disorder. Among the 38 children with ADHD, 13 children had a comorbid learning disorder. The presence of learning difficulties was determined according to the academic aspect of the CDIS. Failing (a score of <60 points on a 100-point scoring system) in at least one core subject among mathematics, Chinese, and English or failing in quizzes in at least one subject three times or more in the recent school year, was defined as academic underachievement in this study. Informed consent was obtained from all children as well as their parents. The complete study was approved by the Ethics Committee of the Peking University Institute of Mental Health in accordance with the Declaration of Helsinki.

	RAN	RAN
	**ADHD**	**TD**
**Regression variables**	F(4,32) = 3.894, P = 0.011	F(4,27) = 2.176, P = 0.099
P1 amplitude	β = –0.082, P = 0.655	**β = 0.461, P = 0.018**
P1 latency	β = 0.070, P = 0.663	β = –0.025, P = 0.899
Age	β = –0.581, P = 0.003	β = 0.237, P = 0.226
IQ	β = –0.053, P = 0.716	β = 0.228, P = 0.243
**Regression variables**	F(4,32) = 6.142, P = 0 .001	F(4,27) = 0.844, P = 0.510
N2pc amplitude	**β = –0.293, P = 0.039**	β = 0.220, P = 0.270
N2pc latency	β = 0.179, P = 0.216	β = –0.099, P = 0.610
Age	β = –0.514, P = 0.001	β = 0.014, P = 0.944
IQ	β = 0.098, P = 0.480	β = 0.165, P = 0.409

Supplementary Table S1. Regression models. ADHD: attention-deficit/hyperactivity disorder; TD: typically developing; RAN: rapid automatized naming
